# Abortion and the Mission of MCH: Perspectives of MCH and Family Planning Professionals in Health Departments

**DOI:** 10.1007/s10995-021-03235-y

**Published:** 2021-10-08

**Authors:** Katie Woodruff, Erin Wingo, Nancy F. Berglas, Sarah C. M. Roberts

**Affiliations:** Advancing New Standards in Reproductive Health (ANSIRH), Department of Obstetrics, Gynecology, and Reproductive Sciences, University of California, San Francisco, 1330 Broadway, Suite 1100, Oakland, CA 94612 USA

**Keywords:** Abortion, Family planning, MCH, Health departments, Professional mission

## Abstract

**Introduction:**

Prior research shows that maternal and child health (MCH) and family planning (FP) divisions in health departments (HDs) engage in some abortion-related activities, largely when legally mandated; some agencies also initiate abortion-related activities. Yet little is known about health department MCH/FP professionals’ views on how abortion-related work aligns with their professional mission.

**Methods:**

Between November 2017 and June 2018, we conducted in-depth interviews with 29 MCH/FP professionals working in 22 state and local HDs across the U.S. We conducted inductive thematic analysis to identify themes regarding participants’ professional mission and values in relation to abortion-related work.

**Results:**

Participants described a strong sense of professional mission. Two contrasting perspectives on abortion and the MCH/FP mission emerged: some participants saw abortion as clearly outside the scope of their mission, even a threat to it, while others saw abortion as solidly within their mission. In states with supportive or restrictive abortion policy environments, professionals’ views on abortion and professional mission generally aligned with their overall state policy environment; in states with middle-ground abortion policy environments, a range of perspectives on abortion and professional mission were expressed. Participants who saw abortion as within their mission anchored their work in core public health values such as evidence-based practice, social justice, and ensuring access to health care.

**Discussion:**

There appears to be a lack of consensus about whether and how abortion fits into the mission of MCH/FP. More work is needed to articulate whether and how abortion aligns with the MCH/FP mission.

## Significance

*What is already known about this topic?* Many maternal and child health (MCH) and family planning (FP) programs in health departments engage in abortion-related activities. Most commonly, MCH and FP programs engage in abortion-related activities that are legally required; some bring public health approaches to legally-required work; and others initiate abortion-related activities based on community need.

*What does this study add?* This study explores how MCH and FP professionals in HDs view abortion in relation to their professional mission. The study finds that only some MCH and FP professionals in health departments see abortion-related activities as part of their professional mission, while others see abortion as solidly outside of their mission. This research suggests that more work is needed to articulate whether and how abortion fits in the MCH and FP missions.

## Introduction

In the field of maternal and child health (MCH), pregnancy is viewed as a unique moment in the life course that provides an opportunity to identify health risks and improve outcomes for pregnant people and their children (Healthy People 2020, 2014). Family planning (FP) is seen as a vital part of the MCH mission, as it helps people time their conceptions and births and supports family wellbeing and attainment of life goals (Sonfield et al., 2013).

Research indicates that accessibility (Penchansky & Thomas, 1981) of abortion care is relevant for MCH populations, but abortion is not often discussed in MCH contexts (Burns et al., 2018). Abortion is a common pregnancy outcome (Jones & Jerman, 2014), and most people who have abortions are already parents (Jerman et al., 2016). Inability to obtain a wanted abortion has adverse effects on pregnant people’s subsequent physical health (Gerdts et al., 2015; Ralph et al., 2019), increases their economic insecurity (Foster et al., 2018b), and has negative effects on the health and development of their existing children (Foster et al., 2018a). Furthermore, people unable to obtain abortions have more health and social service needs (Berglas et al., 2019), which may increase the number of services state and local health departments (HDs) need to provide. This evidence suggests that abortion is relevant to the MCH mission. Yet little is known about how MCH and FP professionals in HDs view abortion-related work.

Research on whether, how, and why HDs engage in abortion-related activities is limited. One study examining websites of state and local HDs found that most state HDs engage in some abortion-related activities; however, these activities largely reflect legal requirements rather than the range of essential public health services (Berglas et al., 2018). The present analysis is part of a broader exploration of whether and how state and local HDs engage in abortion-relation activities. A prior analysis (Berglas et al., 2020) found that HDs engage in abortion-related activities largely when mandated to do so by laws or regulations. However, that analysis also found that some HDs initiate their own abortion-related activities, and some strive to bring public health principles, such as ensuring scientific accuracy, to their legally-mandated abortion work. In interviews, some participants discussed how their approach to abortion-related work was affected by their own sense of professional values and mission as MCH and FP professionals. The current analysis explores this theme to address the question of how MCH and FP professionals in HDs view abortion-related work and its fit (or lack thereof) with their professional mission.

## Methods

Between November 2017 and June 2018, we conducted in-depth interviews with professionals in leadership positions in MCH and/or FP divisions in state and local health departments. We focused on professionals in MCH and FP divisions because these are the health department divisions that focus on public health activities related to meeting people’s pregnancy prevention needs and providing public health services to pregnant people. The overall study included a focus on understanding how health departments’ public health activities related to abortion compared to their other pregnancy-related activities.

### Recruitment

We used purposive snowball sampling, identifying potential participants through professional directories, conferences for state and local MCH/FP leaders, state and local HD websites, and our professional networks. While the senior author asked colleagues in her professional network to refer potential participants, the interviewer did not have preexisting personal or professional relationships with any participants and the senior author only had a preexisting relationship with one of the people who participated in the study. We adjusted recruitment strategies over the study period in order to include a range of geographic regions and to balance state and local HD representation.

We contacted 66 potential participants via email and/or phone to invite participation. Our outreach materials described the study as an exploration of “how [HDs] promote and approach reproductive healthcare and how they engage on abortion specifically. We are especially interested in understanding the challenges and opportunities within [HDs] to facilitate access to abortion services and how this work fits into MCH activities more broadly.” Outreach materials also described our procedures to minimize risk to participants’ confidentiality, including removing all names and other identifiers from audio-recording transcripts, storing transcripts on a secure server, and not connecting participants’ statements with identifying information, rather using regional and broad professional categorizations.

Of those contacted, 22 agreed to participate and completed the interview, 19 declined to participate, and 25 did not respond to our requests. Stated reasons for declining participation included lack of time and/or interest, lack of relevance to their work, unwillingness to take steps to receive approval to participate from superiors, denial of agency permission, and concern over the political implications of the topic.

### Data Collection

Prior to the interview, we sent all participants an information sheet reiterating study aims and confidentiality procedures. The sheet also informed participants of the name and professional title of the interviewer (the second author), but nothing about the interviewer’s or other researchers’ personal background or reasons for doing the research. The interviewer reviewed the information sheet with each participant and confirmed consent to participate verbally at the outset of each interview.

All interviews were conducted by the second author, a research program manager with a graduate degree in public health and formal training in public health theory and principles and qualitative methods. She has experience conducting qualitative interviews on sensitive topics such as substance use, gender-based violence, and discrimination in health care, as well as interviewing professionals about aspects of their work related to reproduction. She has done this work both as an insider and outsider to the communities she is researching. As a public health professional, she shared some part of participants’ professional identity, though she has not worked in HDs.

Our interview guide was semi-structured, covering a pre-determined set of domains but allowing flexibility to explore additional topics that participants thought were relevant. Interview domains included department activities related to family planning, maternal health, and abortion, if any; challenges faced in implementing abortion-related work; and participants’ views on why their department did or did not engage in abortion-related work. The interviewer reiterated our procedures to protect participants’ confidentiality, encouraged them to speak openly, and reminded participants that they could skip any question they wanted to. To elicit frank expression of views, the interviewer used open-ended questions, affirmed that all perspectives were welcome, acknowledged any tensions participants expressed about the controversial nature of the topic, and expressed gratitude for participants’ views.

Interviews were conducted via phone and lasted 30–71 min (mean = 54 min). Each interview was completed in a single session. All interviews were audio-recorded and transcribed. Participants were offered a $50 gift card as a thank you for their participation (though most declined due to their status as government employees). We continued recruiting and interviewing until we obtained sufficient diversity in geographic and state/local HD representation and no new themes emerged from interviews.

The study protocol was reviewed and given an exempt certification by the institutional review board of the University of California, San Francisco, and the research was conducted in accordance with prevailing ethical principles. In reporting results, we followed the COREQ criteria for reporting qualitative research (Tong et al., 2007).

### Analysis

Transcripts were uploaded to Dedoose qualitative data management software. The first and third authors reviewed transcripts and identified excerpts where participants discussed their professional values and mission re: abortion-related activities. The first author coded and analyzed these data using a two-stage process of thematic analysis (Braun & Clarke, 2012): first-round detailed coding to identify a range of concepts arising from the data, and second-round synthesis to consolidate the detailed concepts into broader themes. All authors reviewed all transcripts along with emerging themes and provided input into theme development via group discussions. In analysis, we drew upon existing statements of core public health values (APHA, 2019; Beauchamp, 1976; Lee & Zarowsky, 2015) to identify and synthesize the values respondents described as important to them. We examined whether major themes differed by participants’ professional focus (MCH or FP), type of HD (local or state) and the Guttmacher-rated abortion policy environment in their state, i.e. supportive (“very supportive” and “supportive”), middle-ground (“leans supportive”, “middle-ground”, and “leans hostile”), or restrictive (“hostile” and “very hostile”) (Nash, 2019). In results below, each quote is reported along with the speaker’s professional focus, type of HD, and the abortion policy environment in their state; to protect confidentiality, more specific personal identifiers are not provided.

## Results

Some participants asked colleagues to join them in their interview, so our final sample comprised 29 individual participants across 22 HDs (see Table 1). Participants included directors and assistant directors of MCH and/or FP divisions, and section managers, program directors, and program managers within MCH and/or FP divisions. Ten participants identified themselves as primarily involved in MCH, 14 primarily in FP, and five had responsibilities in both MCH and FP. Most FP programs were nested within an MCH division. HDs were located in states representing all abortion policy environments, although we had no participants from local HDs in restrictive states. We did not collect participants’ gender or other demographic information.

**Table 1:** Interviews conducted, by health department type, U.S. Census region, and state abortion policy environment (n = 22)

	State	Local	Total
Health department type	12	10	22
Census region			
Northeast	3	2	5
Midwest	2	1	3
South	3	2	5
West	4	5	9
State abortion policy environment*			
Supportive	2	5	7
Middle-ground	6	5	11
Restrictive	4	0	4

*Adapted from Nash (2019)

### Mission-Driven Work

Most participants described bringing a strong sense of professional purpose to their work, seeing MCH and FP as mission-driven fields. Participants described their motivating commitment to promoting the health of pregnant or potentially pregnant people and their families. As one participant noted, *Most people who come into family planning are passionate about the subject—so I have a very passionate staff*. (State, FP, supportive policy environment)

In discussing abortion-related work, however, it became clear that participants’ understanding of the scope of their professional mission varied greatly. Some saw abortion as clearly outside the scope of the professional mission of MCH and FP, while others saw abortion-related activities as a critical part of that mission. We explore each of these perspectives.

### Abortion as Outside the MCH/FP Mission

Several participants saw abortion as outside of their professional mission. As one participant noted,

*Our priority is improving pregnancy outcomes, improving women’s access to primary and preventative care. So with that said, I don’t know how abortion fits into that*. (State, MCH, middle-ground policy environment)

Another participant, when asked what work the MCH division was doing related to abortion, replied, *It’s never come up. I’m trying to imagine in what universe that would even come up*. (State, MCH, restrictive policy environment)

#### Seeing Abortion as a Threat to Core Mission

Some participants did not just view abortion as outside their professional scope; they saw abortion as a direct threat to their professional mission. Many participants saw abortion as risky for the health department to engage with:

*[T]here are such strong and passionate feelings on both sides—it would be reckless to run the risk of alienating either side*. (State, MCH, middle-ground policy environment)

Participants perceived that doing abortion-related work would draw unwelcome attention to their programs and potentially threaten their core MCH and FP services:

*I do not want abortion to become the topic that prohibits me from being able to provide the litany of other services that I need to provide. And I cannot win that battle*. (State, MCH, restrictive policy environment)

Another described abortion-related work as *beyond what we’re able to do*, because of a high level of resistance to abortion and corresponding scrutiny of all reproductive health work:

*I mean, it was very difficult to even get family planning contracts approved, and there are a lot of people… who believe that any reproductive health is somehow tied to supporting abortion services. So… it would jeopardize our other funding*. (State, FP, middle-ground policy environment)

#### Framing “Abortion as a Problem” Strategically

Participants who consider abortion to be outside their mission do sometimes engage with the topic of abortion directly, by using policymakers’ discomfort with abortion to mobilize legislators and funders in support of family planning. They frame abortion as a problem, and strategically present family planning as the solution.

*For us, [abortion is] a story-telling tool. So we talk about the decrease in teen birth rates, and the next line is the decrease in teen abortion rates…. It really serves as some nice middle ground for politicians or people who perhaps are more conservative…about family planning and LARC [long-acting reversible contraception]. Once they see some of that abortion data, they’re like “Oh! Okay!”…So those two data points have been very helpful in finding middle ground*. (State, FP, middle-ground policy environment)

Similarly, in describing a challenging political climate for abortion-related work, another participant noted:

*The best that we’ve been able to try to do is to help [the legislature] understand that the best way to reduce abortions is to reduce unintended pregnancies. And the best way to reduce unintended pregnancies is through family planning*. (State, MCH, restrictive policy environment)

In these and other examples, participants made it clear that they frame abortion as a “problem” to be solved, in order to mobilize legislators and funders to support FP services, which they see as central to their core mission.

#### Not Advocating for Abortion-Related Work

Some participants from state HDs noted that their department’s lack of abortion-related work reflected their leadership’s priorities; these participants indicated that their job was to implement what the governor, legislature, or HD leadership decided. Yet the same participants described advocating for programs on other topics. As one described,

*If it’s not [abortion], if there’s a legislative thing that we’re concerned about, oftentimes our legislative liaison… might go talk with the legislature, to see what they’re trying to accomplish and if there’s a different way to do it, to tweak it, or refine it… I would say that is much less often the case with anything related to abortion... It has to do with our internal processing, how problematic it is, or how feasible. It’s very much less likely to happen with any [abortion-related bills.]* (State, MCH, restrictive policy environment)

Another participant said about abortion-related work,

*It’s not a priority, nope. Because our work is governor-appointed, right? We fulfill the wishes and political agenda of the governor… So it’s really up to the governor and to our Medical Director to set the agenda. So I can’t tell you why or why not [abortion is not a priority]*. (State, FP, middle-ground policy environment)

However, this same participant described how the department generated a proposal for a pilot program to provide free LARC services:

*It percolated up through us, from us to the governor’s office. The staff was interested in trying to implement some type of LARC program, and once we vetted it through our process, it [went] to the governor’s office*. (State, FP, middle-ground policy environment)

In other examples of trying to influence the legislative agenda, participants described advocating for comprehensive sexuality education or provision of contraception at school-based health centers. Yet when it came to abortion, they accepted the limits set by the governor or legislature.

### Abortion as Within the MCH/FP Mission

In contrast, other participants viewed abortion a critical health care service and vital to MCH/FP work. One participant described difficult conversations in their department about funding constraints for family planning and abortion, but added, *When we’re having those difficult conversations, what’s not difficult about them is we share the understanding that family planning and abortion are essential primary healthcare and important public health topics that need to be addressed*. (State, FP, middle-ground policy environment)

#### Calling on Public Health Values

Many of these participants directly or indirectly called on core public health values in describing their views on abortion as part of their professional mission. For instance, one referred to accessibility of abortion as an important component of *social justice*:

*I got into doing abortion work because I ultimately feel like it comes down to a justice issue, to me, in terms of abortion being safe and accessible… So I was really excited about working with the health department on that*. (Local, FP, middle-ground policy environment)

Another participant called on the value of *ensuring access to health services*, when describing their frustration with Title X restrictions that kept them from providing in-depth referrals for abortion care as for other care:

*If a patient wants a certain outcome, and that’s their decision [after they’ve] gone through the full amount of counseling, how is that not your job to at least, to the best of your ability to link them to that? …It’s frustrating, because we are able to help with anything else, really, like setting up any other form of appointment and linking them to a resource and a referral—except for abortion*. (Local, MCH, middle-ground policy environment)

The public health value of *evidence-informed policy and practice* was cited by several participants in describing their approach to abortion:

*Even given some struggles [working on abortion], knowing that the [HD] is committed to an evidence-based public health approach is incredibly valuable*. (State, FP, middle-ground policy environment)

#### Collecting Data and Research Evidence to Advocate for Abortion-Related Work

Participants who see abortion as part of their professional mission described their efforts to collect data and research evidence related to abortion and to present it to leadership in order to encourage changes in policy and practice. One participant described how they collected data on abortions and providers in their region in order to advocate for changes to the HD’s referral system:

*In the last year, abortion access has become a topic of conversation…You know, pregnancy numbers are down. Abortion numbers are down... [So] we've had a concentration of providers and concentration of services, because if the numbers get too low, then [providers] can’t maintain services... So [we framed it] in terms of what can we do to maintain services? …[And] to create a stronger referral network locally. Abortion is time sensitive, so how can we create strong referral linkages so that the patients get what they need?* (Local, both MCH & FP, middle-ground policy environment)

Many participants noted that a supportive political climate in their state or locality facilitated their abortion-related work; yet even in more constrained political environments, some participants used evidence to advocate against proposals that would reduce availability of abortion services:

*If we were asking to participate in something around* **increasing** *access to abortion services, I’m almost positive we would be shut down… However, if there’s legislation that proposes to* **restrict** *access to services, then from a public health standpoint, we can actually debate that. We can say that… it has such a significant impact on the population’s health that here are the reasons we’re against this legislation. And we have been supported in crafting those arguments. (State, both MCH & FP, middle-ground policy environment) [emphasis theirs]*

#### Facing Challenges Engaging in Abortion-Related Activities Despite Interest

Participants described several intra-HD challenges they faced in trying to include abortion-related activities in their work. A primary challenge was the need to proactively manage their department leadership, to ensure that abortion is included.

*Just laying the foundation of ‘[Abortion] is a thing we should be including’ and challenging up within the leadership to be thinking about the full range [of reproductive health services]… is really critical*. (Local, FP, middle-ground policy environment)

Participants reported that actively managing leadership requires vigilance and being attuned to leadership’s receptivity to abortion-related work. As one participant described: *You go along, you do your work, you know when you should keep your head down and when you should speak up. You just keep a constant eye on that*. (State, MCH, supportive policy environment)

Another challenge reported by several participants was a need to conceal their department’s abortion-related work: *There is a certain degree of wanting to be somewhat under the radar [about abortion-related activities]… to avoid controversy and negative attention*. (Local, FP, middle-ground policy environment) Another participant described the choice to include abortion in a wider range of services and not put it in the spotlight on its own:

*Any outreach or education we do would be sort of broadly based around reproductive health services, right? And we would include abortion as a sort of core reproductive health service… [We wouldn’t] consider any strategy that sort of solely focuses on abortion. I mean, it just doesn’t make sense both politically and also, just for the need for women to access all sorts of reproductive health services*. (State, FP, supportive policy environment)

Another participant described having to accept cumbersome arrangements related to the organization and operation of delivering abortion clinical care in their local health department, in order to avoid the controversy that would come with the public process of trying to improve these operations.

### Analysis of Themes by Key HD and Participant Characteristics

We compared participants’ perspectives on whether abortion fits within the MCH/FP professional mission by their professional focus, department type, and state abortion policy environment. We found no clear differences in these views based on whether participants were based in an MCH or FP division. More participants from local HDs appeared to see abortion as within their professional mission than participants from state HDs. There appeared to be a relationship between the state abortion policy environment and participants’ views on the role of abortion in the MCH/FP mission: no participants in restrictive state policy environments expressed the view that abortion is within the MCH/FP mission, and no participants in supportive state policy environments expressed the view that abortion is outside the MCH/FP mission. Participants in middle-ground policy environments—where most of our participants were based—were equally likely to see abortion as outside of and included in their professional mission.

## Discussion

In this study, MCH and FP professionals in health departments share a commitment to the mission of promoting the health of pregnant people and their families. Within that commitment, some participants saw abortion as a critical part of the professional mission of MCH and FP, while others saw abortion as outside that scope, even as a threat to their core mission. Several participants in this second group reported that they appealed to policymakers’ perception of abortion as distasteful and/or uncomfortably controversial—in other words, policymakers’ abortion aversion (Roberts, 2019)—in a strategic effort to increase support for family planning services.

Seeing abortion as outside the scope of MCH and FP may be partially explained by the requirements of the federal Title X Family Planning Program and fears of losing Title X funding. Many state and local HDs are Title X grantees (Fowler et al., 2019), with responsibility to fund and oversee local clinics to deliver services for contraception, sexually transmitted infection prevention and treatment, and other preventive care. For decades, Title X required clear separation between its funds and any direct provision of abortion-related services; however, the program also required providers to offer pregnant patients non-directive information and counseling about all of their pregnancy options, including abortion, and to give abortion referrals upon request (Congressional Research Service, 2018). Enforcing these requirements has been a core way that FP professionals in health departments have engaged with abortion (Berglas et al., 2020). In 2019, however, the U.S. Department of Health and Human Services implemented new regulations prohibiting Title X funds from going to any family planning site that also provides or refers for abortion; banning referrals to abortion and mandating referrals to prenatal care; and removing the requirement for all-options counseling that includes abortion (HHS Office of Population Affairs, 2019; Sobel & Salganicoff, 2019). Providers who do not follow these new rules are ineligible to receive federal FP funds. In other words, while our interviews were conducted prior to the Title X regulation changes, those changes demonstrate that participants’ concerns about abortion-related work posing a threat to their core FP efforts were warranted.

However, in response to these new regulations, some health departments declined federal Title X funds (Frederiksen et al., 2019). Thus the Title X changes may reify some MCH/FP professionals’ views of abortion as outside of their mission, but they may also provide new opportunities for professionals to define for themselves whether and how abortion fits into their mission.

We find that MCH/FP professionals’ views on the role of abortion in their professional mission generally align with the state abortion policy environment in which they work. This finding suggests that despite robust evidence demonstrating the adverse MCH health and well-being impacts of pregnant people being unable to obtain abortions (Berglas et al., 2019; Foster et al., 2018a, 2018b; Jerman et al., 2016; Jones & Jerman, 2014; Ralph et al., 2019; Roberts et al., 2014), MCH and FP professionals in health departments may largely approach abortion as a political issue rather than from their professional public health perspectives. By contrast, participants in our study reported willingness to advocate for other controversial reproductive health activities and services, even in some restrictive policy environments. More work to help MCH professionals understand the evidence-based reasons why accessibility (Penchansky & Thomas, 1981) of abortion care impacts MCH populations may help incorporate abortion work into the MCH mission.

Several limitations constrain this work. First, our flexible interview guide meant that we did not ask the same questions of all participants. Therefore, not all participants discussed themes related to professional values and mission. Second, we did not ask about participants’ personal views on abortion. Individual views may color peoples’ perceptions of their work and mission, and we do not know how these views may have influenced the findings, nor how personal and professional views may intersect. Finally, our results are likely affected by selection bias. While we endeavored to balance the sample by geography, more invitees who declined to participate were from Southern states, while more of those who agreed were from the West. Further, we know that some invitees who chose not to participate were concerned about the political nature of the subject matter; in emails declining to participate, some people cited a desire to avoid controversy. Thus this analysis may under-represent the views of MCH/FP professionals from some regions, and those who see abortion as a threat to their core mission.

## Conclusions

This study documents a lack of consensus among MCH and FP professionals in HDs regarding the relationship of abortion to their professional mission. More work is needed to describe whether and how abortion-related work fits into the mission of MCH and FP in health departments.
